# Courtship Behavior of Adult *Spodoptera frugiperda* (Lepidoptera: Noctuidae) Observed Using Track 3D Trajectory Tracking

**DOI:** 10.3390/insects15100824

**Published:** 2024-10-20

**Authors:** Jie Liu, Mariam Tallat, Gensong Wang, Zhi Li, Guoping Li, Xincheng Zhao, Hongqiang Feng

**Affiliations:** 1 Henan Key Laboratory of Agricultural Pest Monitoring and Control, IPM Key Laboratory in Southern Part of North China for Ministry of Agriculture, International Joint Research Laboratory for Crop Protection of Henan, No. 0 Entomological Radar Field Scientific Observation and Research Station of Henan Province, Institute of Plant Protection, Henan Academy of Agricultural Sciences, Zhengzhou 450002, Chinaliguoping1976@163.com (G.L.); 2 College of Plant Protection, Henan Agricultural University, Zhengzhou 450046, China; xincheng@henau.edu.cn; 3 Zhoukou Animal and Plant Disease Prevention and Control Center, Zhoukou 466002, China; zkszbz@163.com

**Keywords:** *Spodoptera frugiperda*, Track 3D, courtship behavior, activity pattern

## Abstract

*Spodoptera frugiperda*, also known as the fall armyworm (FAW), is classified by the Food and Agriculture Organization of the United Nations (FAO) as a major agricultural pest. By gaining a more nuanced understanding of the fall armyworm’s courtship behavior, simpler and more environmentally friendly methods of controlling this pest can be developed. This study used the Track 3D system to meticulously record and describe the activity characteristics and patterns of adult males and females during courtship. The results show that adult FAWs engaged in a variety of activities during courtship that were either discrete (flying, flapping, moving, and crawling), continuous (flapping + flying, flapping + crawling, and flying + crawling), or combined (flapping + touching + flapping; flying + touching + flying). Flying and flapping were the most common activities, with observed flight patterns consisting of parabolic, circular, and zigzag trajectories. The peak activity times for adult FAWs are mainly concentrated at 11:00 p.m., 3:00 a.m., and 5:00 a.m., providing fundamental data for the precise attraction and control of adult FAWs at later stages.

## Simple Summary

This study extended the use of Track 3D trajectory instruments to investigate the previously unobserved nocturnal activities of *Spodoptera frugiperda* (Lepidoptera: Noctuidae), a species of moth Noctuidae. The results indicate that flying and wing flapping are the most common activities, with observed flight patterns including parabolic, circular, and zigzag trajectories.

## 1. Introduction

Research on insect behavior has developed rapidly since 1973 when Frisch, Lorenz, and Tinbergen were awarded the Nobel Prize for their outstanding research in ethology, resulting in a wealth of publications [1]. Insects often engage in courtship before mating, which serves several functions, including confirming the species, sex, and reproductive status of the courting individual, reducing the courting individual’s aggressive tendencies, assessing the quality of potential mates, and coordinating the behavioral and physiological states of both parties [2]. Studies have shown that the mating behavior of most insects exhibits a 24 h rhythm, with moths engaging in courtship and mating activities primarily during dusk and night [3,4,5,6,7]. This temporal rhythmicity may reduce interspecific competition for the same resources and maintain synchrony in mating activities [8]. Traditional insect ethology research has mainly relied on direct observation and manual recording, which are subject to human interference, and insects are not in the most natural state during recording. With advances in technology, numerous commercial digital processing and trajectory analysis software packages have emerged, such as the Track 3D system [9], the MacReflex system [10], and the EthoVision system [11]. The introduction of these software packages has simplified and facilitated the process of digitally processing video images and has greatly advanced the field of insect ethology. The Track 3D system has the advantage over other devices, such as the MacReflex system and the EthoVision system, of being able to accurately track and locate the spatial position of insects in each image. It uses video capture to document insect behavior and automatically synthesizes 3D spatial trajectories. However, a disadvantage of this system is that its camera is not a high-speed camera, which limits its ability to capture finer image data and generate more detailed 3D trajectories.

*Spodoptera frugiperda*, also known as the fall armyworm (Lepidoptera, Noctuidae),is an important agricultural pest that has received global attention from the Food and Agriculture Organization of the United Nations (FAO) [8,12]. After invading China in 2019, the fall armyworm has concentrated its damage mainly on maize, posing a serious threat and a major challenge to safe crop production [13]. Current research on fall armyworm mainly focuses on biological traits [14,15,16,17], integrated pest management [18,19,20], molecular markers [21,22,23,24], and migration patterns [25,26,27]. After eclosion, the primary task of adult fall armyworms is reproduction. Courtship and mating behaviors are important components of their reproductive process and are also a result of their evolutionary adaptation, allowing them to find suitable mates and reproduce successfully in complex environments. Adult fall armyworms are predominantly nocturnal, and relatively little research has been conducted on their courtship behavior. In this study, we used a Track 3D device to record and describe the undisturbed, natural adult behavior of adult fall armyworms. We aimed to understand the intrinsic patterns of courtship behavior by analyzing the trajectories of males and females. In addition, we investigated whether females were active during courtship and, if so, whether it was possible to attract female adult worms with male adult attractants (specific volatiles) to provide data to support upcoming experiments on attracting female *S. frugiperda* with volatiles, which will be critical in facilitating the biological control of female fall armyworms.

## 2. Materials and Methods

### 2.1. Experiment Location

For all experiments, the Track 3D trajectory equipment (Track 3D is a video-based system for the automated tracking of animals in three-dimensional space) (https://www.noldus.com/track3d accessed on 12 March 2017) was used in the laboratory of the Henan Academy of Agricultural Sciences Experimental Base in Yuanyang County, Xinxiang (113°42′ E, 35°0′ N), Henan Province. The temperature in the observation box was maintained at 27 ± 2 °C, and the relative humidity was 65 ± 5%. Continuous recording was performed from 19:00 in the evening to 07:00 in the morning of the following day.

**Figure 1:**
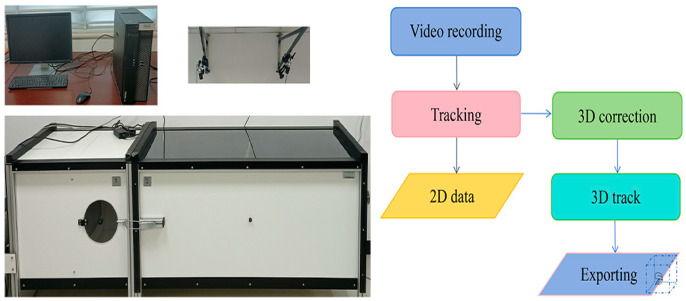
Track 3D system workflow.

A brief explanation of the Track 3D system is as follows: (1) Media recorder: This component is responsible for capturing video footage of the subjects under observation. It records the behavior of the insects in the experimental setup, providing a visual record that can be analyzed later. (2) EthoVision XT 11.5: EthoVision XT is a software package designed to automatically track and analyze animal behavior from video recordings. It processes video data to detect and monitor the movement of subjects within the observation area. (3) CentroidFinder 3D 2.0 Correction: In this step, the CentroidFinder 3D software is used to correct and refine the tracking data. It calculates the exact center of mass (centroid) of the subject in three dimensions, which is essential for accurate motion analysis. (4) Synthesize 3D tracking data: The final step combines all the tracking data into a comprehensive data set. This synthesis allows researchers to analyze subject behavior over time, including patterns, frequencies, and other behavioral metrics. The Track 3D system integrates these components to provide a detailed and automated analysis of insect behavior in a controlled environment. The Track 3D system is shown in Figure 1.

### 2.2. Experimental Material

Plants and Insects: The host plant used in the experiment was maize (Zhengdan 958 variety) at V5–V7 (plant height 45 cm). FAWs were obtained from an artificially reared population at the Institute of Plant Protection, Chinese Academy of Agricultural Sciences, Beijing, China. The larvae were fed on maize leaves under the following rearing conditions: temperature of 26 ± 1 °C, relative humidity of 70 ± 5%, and L:D photoperiod of 16:8 h. After eclosion, the adults were placed in rearing cages with a side length of 30 cm and fed with a 10% honey solution.

Adults in two different reproductive states were used in the experiments: healthy newly emerged unmated adult FAWs and healthy mated adult FAWs. Prior to the start of the experiment, adults and maize were placed in the observation box (160 cm long and 60 × 60 cm wide) of the indoor Track 3D apparatus through the right door of the observation box. All experiments were replicated four times. New individuals were used in each replicate.

### 2.3. Behavioral Observation of Unmated Adult Female FAWs

The experiment was divided into three groups: In the first group, two adults (one female and one male) were placed in the observation box of an indoor Track 3D device; the adult female was allowed to move freely inside the observation box, while the adult male was secured inside a round transparent box, 7 cm high and 8 cm in diameter, with small holes around it. Records from three consecutive nights were considered as one replicate. In the second group, two adults (one female and one male) were placed in the observation box of an indoor Track 3D device, and one adult male was allowed to move freely inside the observation box, while the adult female was secured inside a round transparent box, 7 cm high and 8 cm in diameter, with small holes around it. Records from three consecutive nights were considered as one replicate. In the third group, two adults (one female and one male) were placed in the observation box of an indoor Track 3D device. The adult male and female were allowed to move freely within the box. One night of recording was considered a replicate.

### 2.4. Behavioral Observation of Adult Male and Female FAWs One Day Post-Mating

Adult male and female FAWs were placed in the observation box of an indoor Track 3D device. Adult males and females were allowed to move freely within the observation box. One night of recording was considered a replicate.

### 2.5. Behavioral Observation of Adult Male and Female FAWs Two Days Post-Mating

Adult male and female FAWs were placed in the observation box of an indoor Track 3D device. Adult males and females were allowed to move freely within the observation box, and one night of recording was considered a replicate.

### 2.6. Behavioral Observation of Five Pairs of Unmating Adult Male and Female FAWs

Five pairs of male and female FAWs were placed in the observation chamber of an indoor Track 3D device. The adult males and females were allowed to move freely within the observation chamber.

### 2.7. Data Analysis

Data were analyzed using SPSS 27.0 for analysis of variance and GraphPad Prism 10.0 for graphing. One-way analysis of variance (ANOVA) was used to assess differences in activity frequencies between groups, followed by Duncan’s multiple range test for post hoc comparisons to determine which groups were significantly different from each other. In addition, the activity frequencies of male and female adults were compared using an independent sample t-test to determine if there were significant differences in their activity levels.

## 3. Results

### 3.1. Activity Trajectories of Male and Female Adults

**Figure 2:**
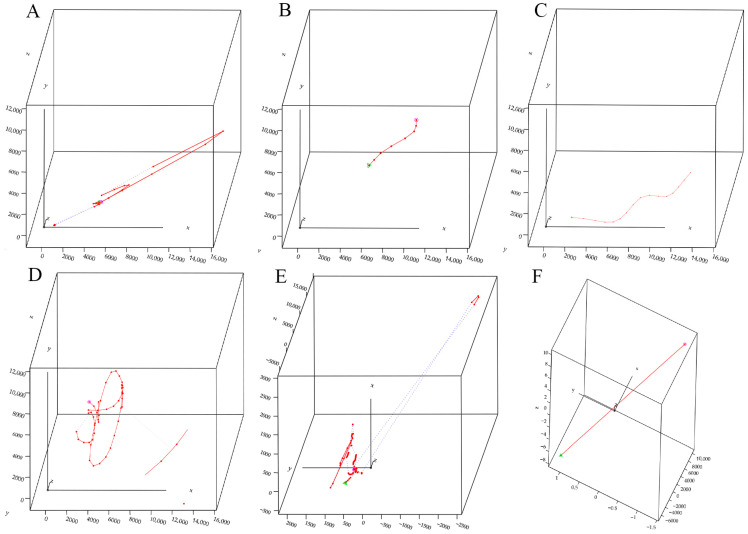
Activity trajectories of adult males and females. (**A**) Return curve flight trajectory of male; (**B**) small arc curve flight trajectory of female; (**C**) large arc curve flight trajectory of adult male; (**D**) circular flight trajectory of female around maize; (**E**) complex chase trajectory of male and female; (**F**) straight flight trajectory of female.

The results indicate that the activity trajectories of male and female adult moths consist primarily of zigzag patterns, circular motions, parabolic trajectories, and linear paths (Figure 2).

### 3.2. The Behavioral Pattern of a Single Adult Female with Free Activity

**Figure 3:**
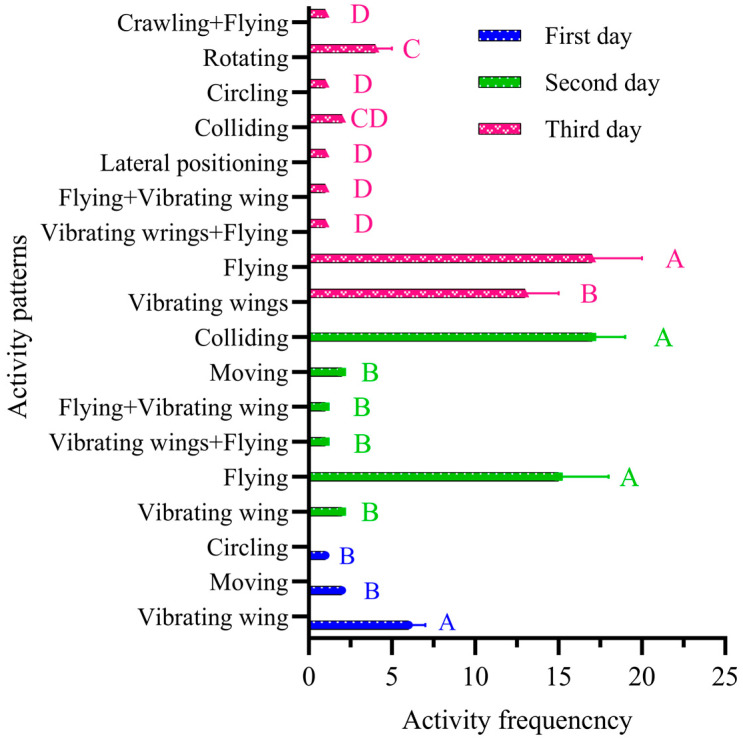
Activity characteristics of single adult female from day one to day three. Data are shown as mean ± SD. Capital letters above bars indicate significant differences in activity frequency between days.

The results indicate that on the first day, the adult female fall armyworm exhibited only three patterns of activity, with only nine activities recorded. The total duration of these activities was 30 s, and each activity lasted in the range of 1~7 s. The behavioral characteristics were predominantly wing flapping (six times) (66.67%), followed by moving (two times) (22.22%) and circling (one time) (11.11%) (Figure 3).

On the second day, the adult female fall armyworm exhibited six different activity patterns with a total activity frequency of 38 times. The cumulative activity duration was 51 min and 4 s, with individual activities lasting in the range of 1~766 s. Predominant behaviors included colliding with the observation chamber glass 17 times (44.74%) and flying 15 times (39.47%). The behavior of first flapping and then flying and the behavior of first flying and then rapidly flapping wings each occurred once (2.63%). Both flapping and moving occurred twice (5.26%) (Figure 3).

On the third day, the adult female fall armyworm was more active with a total of 41 activities recorded. The total duration of these activities was 352 s, and the duration of individual activities ranged from 1 to 181 s. The main behaviors were flying and flapping, which occurred 15 and 13 times, respectively. During flying activities, the female made contact with the host plant seven times. Other behaviors observed included colliding with the observation chamber glass twice, flapping followed by flying once, flying followed by rapid flapping once, crawling followed by flying once, turning five times, and sideways swiping once (Figure 3).

### 3.3. The Behavioral Pattern of the a Single Adult Male with Free Activity

**Figure 4:**
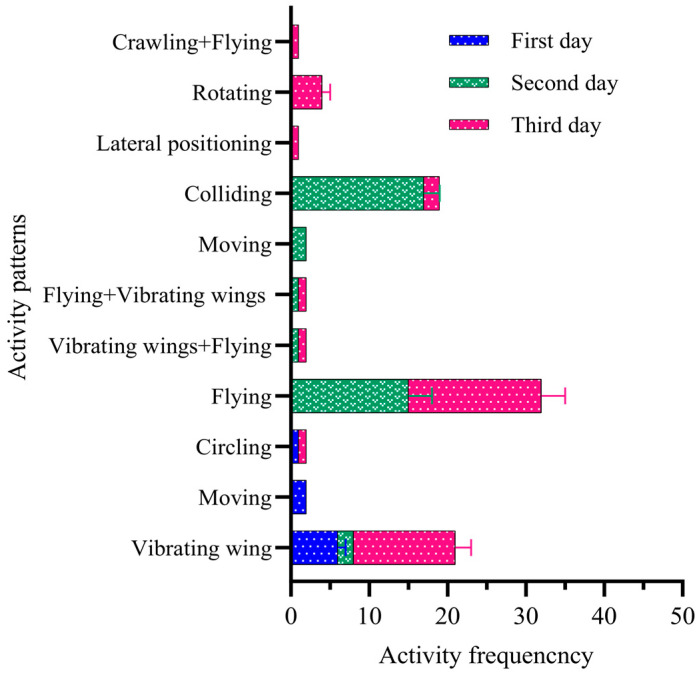
Activity characteristics of single adult male from day one to day three. Data are shown as mean ± SD.

The results indicate that the adult males exhibited relatively little activity on the first day, with only 12 instances of movement for a total of 0.23 h. The duration of each activity was in the range of 5~54 s, with peak activity being observed between 12:00 a.m and 1:00 a.m. The activity patterns included two main types: crawling first and then flying, which occurred nine times (75%), and flying, which occurred three times (25%). During the activity process, the female landed on the maize plant four times (Figure 4).

On the second day, the adult male fall armyworm exhibited a total of 42 activities with a total activity duration of 0.32 h. The duration of each activity ranged from 9 to 59 s. Behavioral characteristics were predominantly flying and crawling, with specific sequences of these activities also being observed. The male performed flying activities 35 times (83.3%) and crawling activities 5 times (11.9%). The behaviors of first crawling and then flying and first flying and then crawling occurred once each (2.4%) (Figure 4).

On the third day, adult fall armyworm activity was quite frequent, with a total of 47 activities recorded. The cumulative activity duration was 0.39 h, and the duration of each activity ranged from 3 to 46 s. The behavioral characteristics were mainly flying, crawling, and moving, with specific sequences of these activities also bring observed. There were 33 instances of flying (74.21%), 2 instances each of crawling and flying followed by crawling (4.26% each), and 7 instances of moving (17.89%). The behavior of crawling followed by flying occurred three times (6.38%). During the activity process, there were three times (peak activity period) when the adult FAW landed on the maize plants (Figure 4).

### 3.4. The Patterns of Free Activity in Adult Females and Males

**Figure 5:**
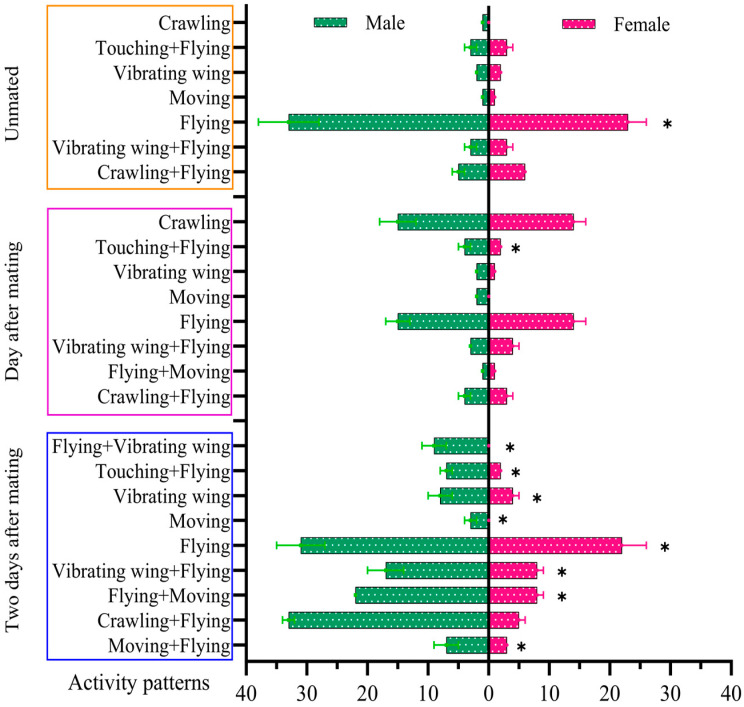
Patterns of adult male and female FAWs with free activity. Differences in activity frequency between female and male were analyzed by *t*-test. * indicates statistically significant differences (*p* < 0.05) in activity frequency between male and female.

The behavior of adult males and females consisted mainly of two patterns, active and resting. The total number of activities for adult males and females was 115. The total activity time was 1.07 h. The adult female had a total of 50 activities (43.48%), and the adult male had 65 activities (56.52%), with a significant difference between them (*p* < 0.05; df = 6; t = −4.825). Eight different activity patterns were observed, with the most frequent activities being flying and flying followed by crawling. Flying occurred 76 times (66.09%), with flight durations in the range of 1~491 s. Crawling was observed eight times (6.96%), with crawling durations in the range of 1~64 s. Crawling followed by flying occurred 19 times (16.52%), with durations in the range of 1~167 s (Figure 5).

### 3.5. Patterns of Adult Male and Female FAWs One Day Post-Mating

The total activity frequency of male and female adults was 222 times, with a total activity duration of 0.96 h and individual activities lasting in the range of 1~55 s. In addition to single activity behaviors, the male and female FAWs exhibited sequential or interleaved activities. This included nine different sequential movement patterns. Flying was the main behavioral activity (113, 50.91%) with 13 instances of flying around the host plant and flight durations in the range of 1~55 s. This was followed by crawling (27; 12.10%) and patting (25; 11.26%) with a ratio of rapid to regular flapping of 40% to 60% and flapping durations in the ranges of 3~29 s and 1~55 s, respectively (Figure 5).

### 3.6. Patterns Adult Male and Female FAWs Two Days Post-Mating with Free Activity

The total frequency of activities of male and female adults was 13 times, and the total duration of activities was 131 s. There were two modes of activities, flight and crawling. The number of flights was 10 times (61.83%), with a total flight time of 81 s and a duration of 1~39 s. The number of crawls was three times (87.5%), with a duration of 3~40 s (38.17%) (Figure 5).

### 3.7. Patterns of Five Pairs of Adult Male and Female FAWs with Free Activity

**Figure 6:**
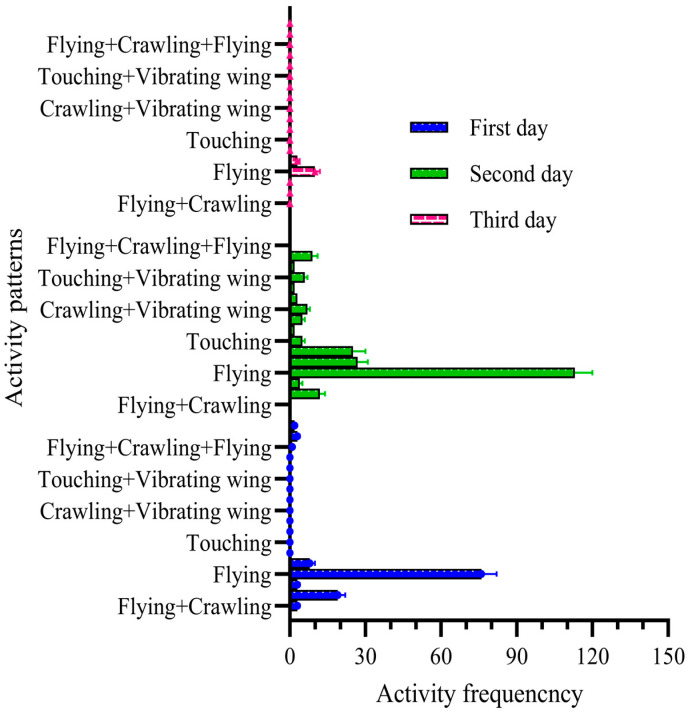
Frequency of different activity patterns in paired adults from day one to day three.

The results show that on the first day, the total number of activities for female and male adult FAWs was 52 times (39.37%) and 87 times (62.59%), respectively. The total activity duration of males was 1.68 times that of females (t = 9.468; df = 6; *p* < 0.05). The females and males performed seven and nine different types of activities, respectively. The duration of each activity was in the range of 3~39 s for females and 3~50 s for males. (Figure 6). On the second day, the total numbers of activities for adult female and male FAWs were 39 times (45.88%) and 46 times (54.12%), respectively. The total duration of activity for males was 1.42 times that of females (t = −2.425; df = 6; *p* > 0.05). The females and males performed seven and eight different types of activities, respectively. The duration of each activity was in the range of 4~38 s for females and 3~55 s for males. On the third day, the total numbers of activities of male and female adults were 38 times (44.19%) and 48 times (55.81%), respectively, the total activity duration of male adults was 1.14 times longer than that of females (t = −2.2.97; df = 6; *p* < 0.05), the numbers of activity behaviors of males and females were six and seven, and the activity duration of males and females were in the ranges of 2~40 s and 4~53 s, respectively. The duration of activity was in the ranges of 2~40 s and 4~53 s for males and females, respectively (Figure 6).

### 3.8. The Behavioral Patterns and Specific Descriptions of a Pair of Adult Male and Female FAWs during Courtship

**Figure 7:**
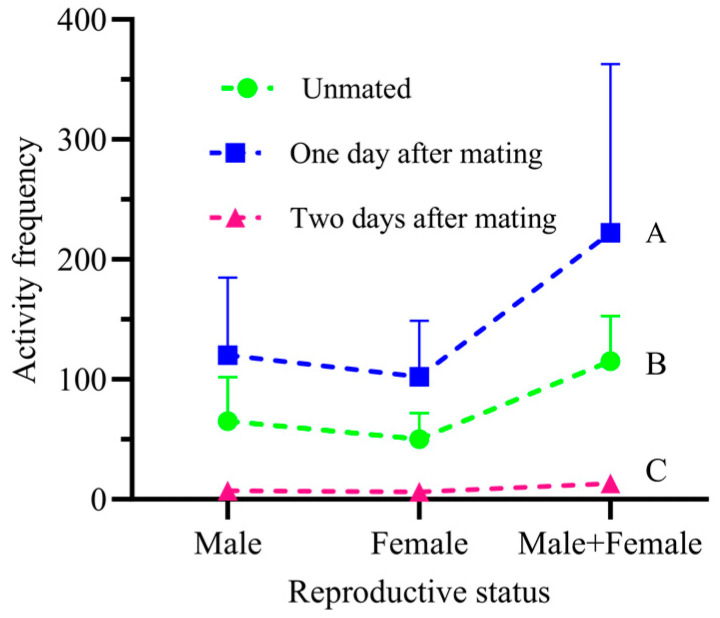
Total activity times of pair of male and female adult FAWs at different reproductive stages. Capital letters above bars indicate significant differences in total activity frequency between male and female adults at different reproductive stages.

A pair of adult male and female moths moving freely in the observation chamber showed differences in total activity frequency at three different reproductive stages: unmated, one day after mating, and two days after mating (*p* < 0.05; df = 2; F = 1310.68). Unmated, the adult female had a total of 50 activities (43.48%), and the adult male had 65 activities (56.52%), with a significant difference between them (*p* < 0.05; df = 6; t = −4.825). One day after mating, the adult female had a total of 102 activities (45.95%), and the adult male had 120 activities (54.05%), again with a significant difference (*p* < 0.05; df = 6; t = 7.675). Two days after mating, the adult female had a total of six activities (46.15%), and the adult male had seven activities (53.85%), with no significant difference between them (*p* > 0.05; df = 6; t = −2.782) (Figure 7).

Mating behavior was observed in all three distinct reproductive states. During initial courtship, the male physically touched the female with his body. The female’s most direct method of rejection is to fly away quickly, and this entire process is very quick, lasting only 8 s. The second courtship event is relatively longer, with a total duration of 13 min 45 s, including 4 min 3 s of activity and 17 activity occurrences. Touching between the male and female during courtship may be repeated several times. After the male touches the female, she may move backwards and sometimes vibrate her wings. The third courtship event lasts a total of 13 min and 20 s, with an activity duration of 6 min and 9 s with five activity occurrences, and a rest duration of 6 min and 49 s with four rest occurrences.

### 3.9. Activity Rhythm of Adult Male and Female Fall Armyworms

**Figure 8:**
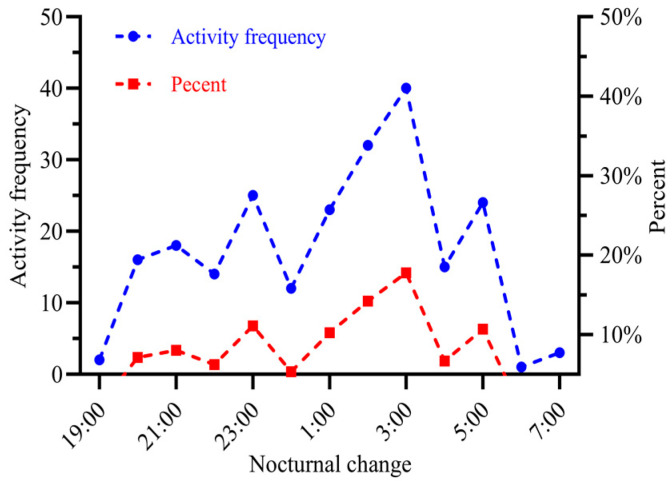
Nocturnal changes in the frequency of activity of adult FAWs.

Adult FAWs begin their activities at 19:00 h, and these activities typically end around 7:00 h the following day. From 19:00 to 21:00, there is a gradual increase in activity as the insects reach their first peak period. Later, between midnight (12:00 a.m.) and 3:00 a.m., adult activity gradually intensifies, marking the second peak period. During the early hours of the next day, from 5:00 a.m. to 7:00 a.m., the frequency of adult activity decreases over time. Peak activity periods for adult FAWs are primarily concentrated around 11:00 pm, 3:00 am, and 5:00 a.m. Rest periods for these adults are primarily concentrated at 10:00 p.m., midnight (12:00 a.m.), 4:00 a.m., and 6:00 a.m. (Figure 8).

## 4. Discussion

Insects exhibit a variety of behaviors, some of which are instinctive, with some behaviors often showing periodicity and rhythmicity [1]. In this study, the Track 3D device was used to analyze the activity characteristics and patterns of male and female adult FAWs in both solitary and mixed modes. The study categorized adult FAW behaviors into two main types: activity and rest. The observed behaviors included three forms, namely single activities, sequential activities, and interleaved activities, for a total of 14 different activities. The most frequent and prolonged activities were flight-related, which is in line with the species being a long-distance migratory insect [27]. On the first day, both male and female activities were relatively infrequent in single modes. However, on the following day, there was an increase in both frequency and pattern of activity. Both sexes exhibited wing flapping behavior, which may be used to attract the opposite sex from the environment [28], an essential visual component of male courtship [29]. Rapid wing vibration may facilitate the release of volatiles or produce auditory cues. The most significant difference in behavioral activity between male and female adults was observed on the second day in solitary mode, with female adults primarily engaging in colliding behavior and males focusing on flying. The decrease in colliding behavior in female adults on the following day could be an adaptation to the environment, and the specific reasons for this need to be further investigated. In addition, on the first day in solitary mode, female adults were relatively less active and spent most of their time in a resting state. Male and female adult moths circle and spiral over the fixed opposite sex. Combining findings from previous studies stating that adults can mate several times in their lifetime, this study found that a pair of male and female adult moths had different overall activity frequencies in three different reproductive states. These differences may be related to their reproductive behavior and circadian rhythms, suggesting that the reproductive state of male and female adults may influence their behavior.

Once the fall armyworm enters the adult stage, all life activities revolve around reproducing offspring, and adult males and females may mate multiple times [17], which is consistent with the results of this study. The combination of crawling and flying may indicate a multimodal approach to resource location, and the sequence of these activities (e.g., crawling followed by flying and vice versa) may reveal specific behavioral conventions. Crawling and flying also directly or indirectly affect communication in some way [30]. In the Pentatomidae, wing flapping for communication is common and mediates short-range communication during courtship [31]. The flapping activity of adult FAW can be divided into two types: regular flapping and rapid flapping. Regular, low-frequency flapping may be associated with flight practice, whereas rapid flapping during courtship may be associated with mate recognition mechanisms. Wing flapping followed by flight as a special behavior may be associated with courtship, mating rituals, or defensive maneuvers. It is noteworthy that wing flapping-related behaviors are more common prior to the day of courtship, suggesting that they may play a role in establishing territory or attracting mates [31].

Insects exhibit a variety of complex behaviors that are influenced by environmental cues, increasing their adaptability to different environments [32]. During courtship, adult female moths are also very active with a high frequency of activity. This suggests that female moths play an important role in mating. The peak activity times for adult FAWs are mainly concentrated at 11:00 pm, 3:00 a.m., and 5:00 a.m., providing fundamental data for the precise attraction and control of adult FAWs at later stages. In addition to the 14 active behaviors mentioned above, male and female adult FAWs also exhibit resting behaviors, suggesting that they, like other insects, have certain ecological and physiological needs. This is true regardless of their life history and ecology [18,33,34,35,36,37]. In female insects, mating can alter a range of behaviors, including feeding, movement, sleep, oviposition, and visual response in addition to life span [38,39]. In ants, changes in vision, circadian rhythm, and phototransduction occur after mating [40,41]. A decrease in activity frequency after mating in adult male and female FAWs suggests that their reproductive state has some effect on behavior.

Sexual selection has led to diversity in courtship displays. In insects, courtship involves complex and highly variable behaviors across multiple sensory modalities. Notable examples include the songs and pheromones of crickets [42,43] and the vivid bioluminescent displays of fireflies [44]. Sexual communication in butterflies involves the use of different signals at different stages of mate recognition and selection, resulting in a complex scenario [45]. In this study, we found that males actively fly around suitable areas in search of females and approach the females they encounter. This suggests that males take an active role in the courtship process, while females are more passive. Movement and circling activities were also observed in mixed modes, which may indicate exploratory behavior and habitat assessment [30]. On the day of courtship, adults made the most frequent contacts with the host plant, maize, suggesting that FAWs frequently fly to their host plant in search of mates. This phenomenon is common in entomology, especially among insects that depend on specific plants for reproduction. Insects use flight to expand their range in search of mates, which also helps them find suitable host plants for oviposition.

Insects possess several different types of circadian rhythms [46], including movement/flight [47,48], which may directly or indirectly influence pheromone communication in certain aspects. For each species occupying different ecological niches, the onset, peak, and duration of circadian rhythms during the light or dark phases are different. Studies have shown that the mating activities of most insects exhibit a 24 h behavioral rhythm [3,5,6], with moths mating primarily at dusk and dawn [7]. This temporal rhythmicity in mating activities may reduce competition between species for the same resources and maintain synchrony in mating activities [3]. The results of this study indicate that the peak activity times for adult FAW are primarily concentrated at 11:00 p.m., 3:00 a.m., and 5:00 a.m. This suggests that making the right decisions at the right time is critical for survival, especially when searching for food, mates, or breeding sites or escaping predators in complex and dynamic environments [49].

## Data Availability

Data are available upon request from the authors.
